# Evaluation of the Relationship Between Clinical Symptoms and Modic Changes

**DOI:** 10.7759/cureus.6970

**Published:** 2020-02-12

**Authors:** Serdar Çevik, Hakan Yılmaz

**Affiliations:** 1 Neurosurgery, Memorial Şişli Hospital, İstanbul, TUR; 2 Radiology, Uşak University, Uşak, TUR

**Keywords:** low back pain, magnetic resonance imaging, modic changes, spine

## Abstract

Purpose

The aim of this study was to evaluate the association between changes in the type of Modic change (MC) and newly developed MCs and low back symptoms.

Methods

This retrospective study includes all the patients suffering from low back pain who has at least two magnetic resonance studies between 2013 and 2016. Patients with MC in at least one vertebra in magnetic resonance imaging (MRI) images were included in the study. The patients who have periods of two MRIs less than six months were excluded.

Results

A total of 129 patients were included in the study and 774 vertebrae were evaluated. At the time of the first admission, MC was observed in 283 out of the 774 vertebrae (37%), whereas in the second admission, MC was observed in 343 of 774 (43%) vertebrae. New MCs were observed in 59 vertebrae. Two patients showed complete resolution of MC in the second admission. It was observed that patients with type 1 change were more associated with pain than other types (p=0.001).

Conclusion

In our retrospective case-control study, we have found that MCs can transform into different types or disappear completely over time. A significant positive correlation was found between the presence of MC type 1 changes in the visual analog scale(VAS).

## Introduction

It has been suggested that Modic changes (MCs) are a specific reason for low back pain (LBP) [1-3]. In recent years, it has started to be a subject that attracts attention. MCs have been determined by magnetic resonance imaging (MRI) since 1988 (Modic et al).; based on histological studies, they are divided into three types [4]. A type I MC shows a signal reduction in T1-weighted (T1w) MRI and a signal increase in T2-weighted (T2w) MRI, and, histologically, reflects inflammatory changes in the vertebral endplates [4]. A type II MC T1w and T2w show both signal increase and represent a more stable phase of degenerative disc disease but has the potential to transform into another type. A type III MC shows decreased signal intensity in both T1w and T2w and is associated with large subchondral bone sclerosis in plain radiography scans [4-5]. However, when an MC type is transformed into another, mixed MC types can develop [1,4,6-7].

The relationship between MC and LBP has been studied many times, and the presence of MC has been reported to increase pain levels [8-10]. MC type II may depict a more stable phase of degenerative disc disease but has the potential to transform into another type [11-14]. However, type 1 MC has been shown to be more correlated with LBP symptoms than other varieties in both the general population and the clinical patient group [3,15-18]. MCs are quite common in patients with LBP, but the long-term clinical effects have not yielded sufficient results. The aim of this study was to evaluate the association between changes in the type of MC, newly developed MCs, and low back symptoms.

## Materials and methods

This retrospective study includes all the patients suffering from low back pain who have had at least two magnetic resonance studies between 2013 and 2016 in Ağrı state hospital. Patients with MC in at least one vertebra in MRI images were included in the study. The patients who have had at least two MRIs at less than six months were excluded. Patients who had vertebral fractures, spondylolisthesis (≥4 mm), spinal stenosis, disc extrusion, neoplasia, inflammatory vertebrae, lumbar disc herniation or lumbar stabilization were not included in the study. Annular tears, disc bulging, and facet joint degeneration were excluded. Because they are often associated with pain and creates biases about the source of low back pain.

The VAS pain score of the 129 patients in the hospital data system was examined. The VAS scores were defined as 0 indicating no pain and 10 indicating the worst pain. This study was approved by the local ethics committee and informed consent was obtained from each participant.

MR imaging method and image analysis

All MR images were obtained using a 1.5T scanner (Siemens, Munich, Germany, or Philips, Best, The Netherlands). For the present study, L1-S1, T1- and T2-weighted sagittal, and T1-weighted axial MRI images were acquired using the following protocol: slice thickness, 5 mm; slice gap, 1 mm; field of view, 280 × 240mm^2^; and matrix,448 × 336.

All MR images were analyzed by a radiologist who was not informed about the clinical characteristics of patients. The initial and second admission MR images of the patients were evaluated from the L1 corpus to the S1 corpus according to the criteria determined in previous studies [13]. T1 and T2 weighted sagittal images were evaluated, and the modular changes were reported as previously described in the literature (MC type I, MC type II, MC type III, MC type I / II, MC type II / III MC type I/II/III) [5].

Descriptive statistics were used for continuous variables (mean, standard deviation, minimum, maximum, median). The student's t-test was used to compare VAS scores among the Modic group. Significance was set at the 5 % level and all analyses were performed using IBM SPSS Statistics version 22 (IBM Corp., Armonk, NY).

## Results

**Figure 1:**
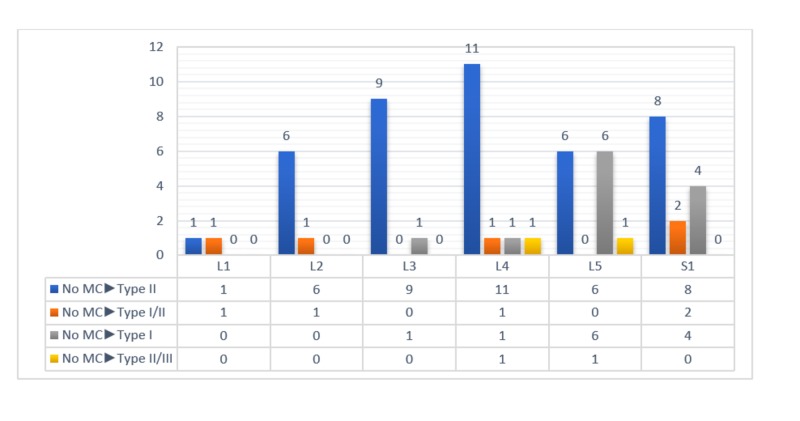
The distribution of the newly developed MCs at the time of second admission and their distribution according to MC types MC: Modic change

**Figure 2:**
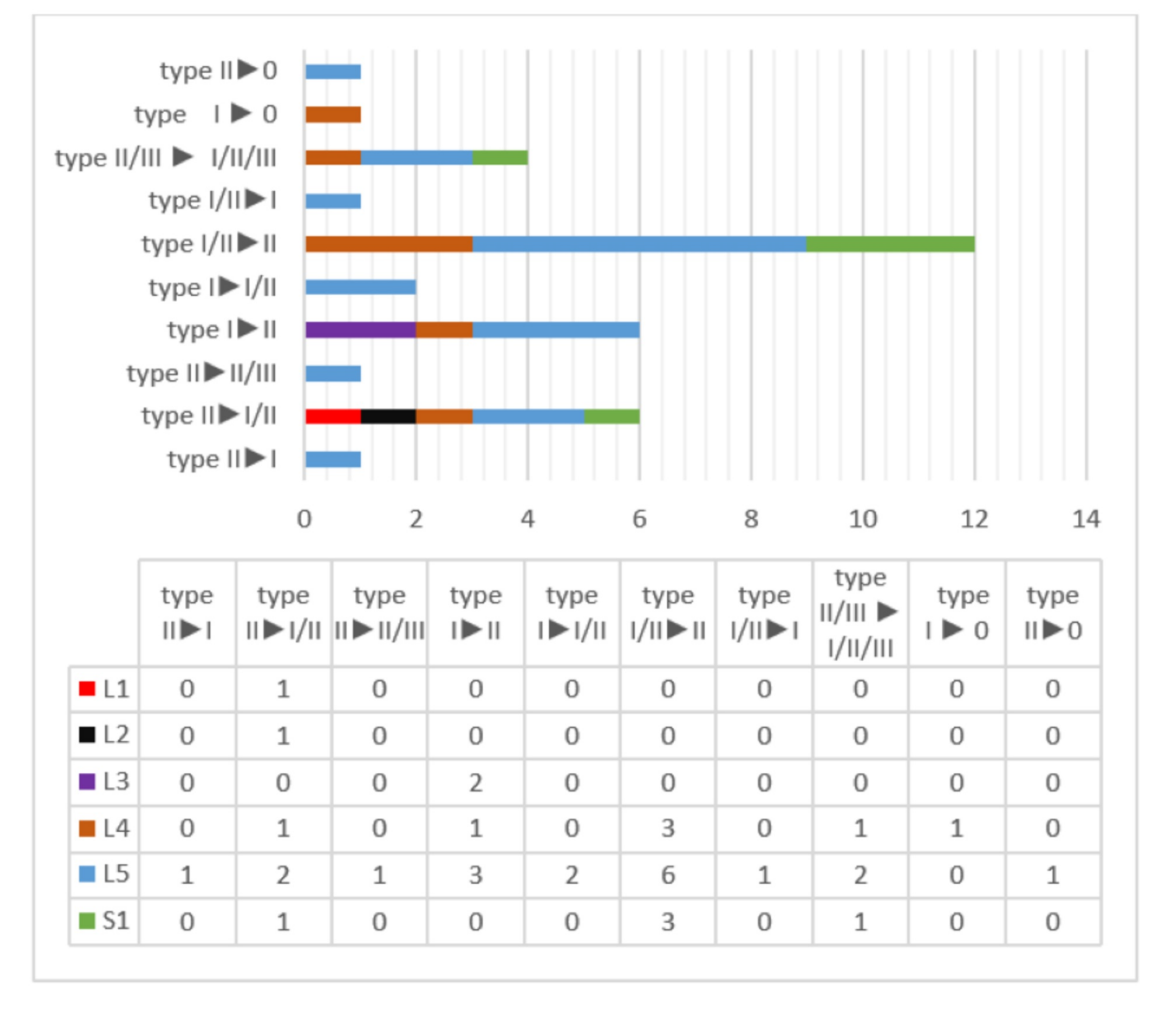
Transformation of MCs in the second admission according to vertebra levels and MC types MC: Modic change

A total of 129 patients (774 vertebrae) (n = 91 (67%) females and n = 38 (33%) males) were included in the study. The mean age of the patients was 42 (min. 18 - max. 77) and the mean time between the first and second admissions was 13.3 months (6-24 months). At the time of the first admission, MC was observed in 283 of the 774 (37%) vertebrae, whereas in the second admission, MC was observed in 341 of 774 (43%) vertebrae. The first and second admissions' scanning results are shown in Table 1. New MCs were observed in 60 vertebrae (Figure 1). Twelve of these cases had type I, 41 had type II, five had type I / II, and two had type II / III. MCs were observed to be transformed in 35 vertebrae (Figure 2). MCs disappeared completely in two patients (Figure 2).

**Table 1:** Distribution of MCs according to vertebral level in 129 patients (774 vertebrae in total) MC: Modic change

	First Admission						Second Admission				
	type I	type II	type I/II	type II/III	type I/II/III	type I	type II	type I/II	type II/III	type I/II/III
L1	0	8	0	0	0	0	9	1	0	0
L2	0	22	0	0	0	2	27	2	0	0
L3	5	39	2	0	0	2	49	3	0	0
L4	2	56	7	2	0	1	70	6	1	1
L5	5	66	14	2	0	8	76	11	3	2
S1	0	45	7	1	0	4	55	7	0	1
total	12	236	30	5	0	17	286	30	4	4

The mean VAS score was 5.44 (SD 1.26, range 3-9) at the time of the first admission and the mean VAS score was 5.56 (SD 1.2, range 3-9) at the second admission. In the second admission, the mean VAS score of 12 patients with the new MC type 1 was 6.71 (SD 1.32, range 4-9) while the mean VAS score was 5.14 (SD 1.09, range 4-9) at the first admission (p = 0.003). The mean VAS score of patients with MC type I and M type I / II changes at the time of the first and second admission was 6.62 (SD 1.24, range 4-9) and 6.65 (SD 1.12, range 4-9) and the mean VAS score of patients without MC type I changes at the time of the first and second admissions was 5.12 (SD 1.07, range 3-7) and 5.29 (SD 1.06, range 3-8, respectively; (p = 0.001, p = 0.001, respectively).

## Discussion

In the present study, we found that the transformation rate in the 13 months' mean period MCs was 12.3%. A significant positive relation was found between the development of new MC type I and transformation to MC type I from other types and the severity of pain. The transformation from MC type I to MC type II was related to a decrease in pain, but it is not statistically significant.

The present study shows that all evaluated MCs can be converted into almost any type and that they can completely disappear and not persist in follow-up (Figure 2). In our study, it was seen that 33% of vertebrae from type I / II to type II and 17% from type I to type II. However, 17% of the vertebrae were seen from MC type I / II to MC type I and 3% from MC type II to MC type I. In one patient, MC type I at L4 level in one patient, and MC type II changes at L5 level at one patient disappeared during the follow-up period. This situation does not comply with the assertion that MCs can be transformed from type I to type I / II or type II [1,4,6-7]. However, in the 14-month follow-up study conducted by Jensen et al. in 2012, they have found conflicting results with the developmental path hypothesis, as in our results [17].

The present study shows a statistically significant relationship between MC type I and the level of LBP. The VAS score of patients with MC type I at the time of first admission and second admission was significantly higher than the other types. In the recent two cohort follow-up studies, patients with MC type I at both first admission and follow-up were found to have a worse prognosis as compared to other types, which was consistent with that in our study [17,19]. The control VAS score was found to be statistically significantly higher than the first admission VAS score of patients with new MC type I (20%) and type I / II (8%) in the second control. Therefore, our findings highlight that MC type I has a stronger relationship with LBP than other MC types [4,10-11,13,20-22]. It is not clearly known how MCs cause pain. However, in human and animal studies, vertebral endplates are known to have immune reactive nerves [23-24]. When the endplate histology of patients with discogenic LBP was evaluated, more inflammation and axon growth were detected in the endplate with MC than the normal endplate cases [25]. In addition, in a one-year follow-up study of chronic LBP patients, the development of MC type I and the increase in the vertebral involvement of the current MC type I showed that adjacent disc degeneration accelerated and disc degeneration was slowed down in patients without MC type I, which suggests that MC type I may be more related to LBP symptoms [26].

There are some limitations to this study. This study did not evaluate other degenerative imaging findings (i.e., disc degeneration, disc bulging, and Schmorl’s nodes). It is based on the assumption that the only source of pain in LBP patients may be MCs. The potential effect of other degenerative MRI scan analyses of LBP symptoms, pain medication, somatic and psychological comorbidities, educational level, and other psychosocial factors have not been evaluated.

## Conclusions

In our retrospective study, we have found that MCs can transform into different types or disappear completely over time. MC type I has a stronger relationship with LBP than other MC types.
